# Perivascular spaces in the centrum semiovale at the beginning of the 8th decade of life: effect on cognition and associations with mineral deposition

**DOI:** 10.1007/s11682-019-00128-1

**Published:** 2019-06-27

**Authors:** Maria del C. Valdés Hernández, Lucia Ballerini, Andreas Glatz, Susana Muñoz Maniega, Alan J. Gow, Mark E. Bastin, John M. Starr, Ian J. Deary, Joanna M. Wardlaw

**Affiliations:** 1 Department of Neuroimaging Sciences, Centre for Clinical Brain Sciences, University of Edinburgh, 49 Little France Crescent, Chancellor’s Building, Edinburgh, EH16 4SB UK; 2 Dementia Research Institute, University of Edinburgh, 49 Little France Crescent, Chancellor’s Building FU-427, Edinburgh, EH16 4SB UK; 3 Centre for Cognitive Ageing and Cognitive Epidemiology, Department of Psychology, University of Edinburgh, 7 George Square, Edinburgh, EH8 9JZ UK; 4 Department of Psychology, School of Social Sciences, Heriot-Watt University, Edinburgh Campus, David Brewster Building (Room 2.63A), Edinburgh, EH14 4AS UK; 5 Alzheimer Scotland Dementia Research Centre, Department of Psychology (Room G24), University of Edinburgh, 7 George Square, Edinburgh, EH8 9JZ UK; 6 Department of Psychology, University of Edinburgh, 7 George Square, Edinburgh, EH8 9JZ UK; 7 Row Fogo Centre for Ageing and the Brain, University of Edinburgh, 49 Little France Crescent, Chancellor’s Building, Edinburgh, EH16 4SB UK

**Keywords:** Iron deposition, Brain, Perivascular spaces, Virchow-Robin spaces, MRI, Cognition, Ageing

## Abstract

Brain iron deposits (IDs) are indicative of microvessel dysfunction which may predispose to small vessel disease (SVD) brain damage and worsen cognition later in life. Visible perivascular spaces in the centrum semiovale (CSO-PVS) are SVD features linked with microvessel dysfunction. We examined possible associations of CSO-PVS volume and count with brain IDs and cognitive abilities in 700 community-dwelling individuals from the Lothian Birth Cohort 1936 who underwent detailed cognitive testing and multimodal brain MRI at mean age 72.7 years. Brain IDs were assessed automatically followed by manual editing. PVS were automatically assessed in the centrum semiovale and deep corona radiata supraventricular. General factors of overall cognitive function (g), processing speed (g-speed) and memory (g-memory) were used in the analyses. Median (IQR) volumes of IDs and CSO-PVS expressed as a percentage of intracranial volume were 0.0021 (0.011) and 0.22 (0.13)% respectively. Median count of CSO-PVS was 410 (IQR = 201). Total volumes of CSO-PVS and ID, adjusted for head size, were correlated (Spearman ρ = 0.13, *p* < 0.001). CSO-PVS volume, despite being correlated with all three cognitive measures, was only associated with g-memory (B = -114.5, SE = 48.35, *p* = 0.018) in general linear models, adjusting for age, sex, vascular risk factors, childhood intelligence and white matter hyperintensity volume. The interaction of CSO-PVS count with diabetes (B = -0.0019, SE = 0.00093, *p* = 0.041) and volume with age (B = 1.57, SE = 0.67, *p* = 0.019) were also associated with g-memory. Linear regression models did not replicate these associations. Therefore, it does not seem that CSO-PVS burden is directly associated with general cognitive ability in older age.

**Electronic supplementary material:**

The online version of this article (10.1007/s11682-019-00128-1) contains supplementary material, which is available to authorized users.

## Introduction

It is known that minerals accumulate in several brain regions and cell types (Ward et al. 2014). Macro-aggregation of minerals with para−/ferromagnetic properties is distinguishable on T2*-weighted gradient echo and susceptibility-weighted (SWI) magnetic resonance imaging (MRI) (Valdes Hernandez et al. 2012a). The mineral most commonly found in these aggregates is iron. It mainly accumulates gradually with age in areas associated with motor activity (Rouault 2013) (i.e. corpus striatus, substantia nigra and brainstem) via dysfunctional brain regulatory mechanisms (McCarthy and Kosman 2014), or as a residual from very small chronic haemorrhages, namely microbleeds, in abnormal blood vessels (i.e. capillaries) throughout the brain (Martinez-Ramirez et al. 2014). The iron deposits (IDs) coming from these two sources can aggregate with other minerals forming “calcified” clusters (Ramonet et al. 2002; Valdes Hernandez et al. 2016; Valdes Hernandez et al. 2014). Several MRI studies have reported associations of these three forms of mineral accumulations (Yates et al. 2014) with cognitive decline (Valdés Hernández et al. 2015; Sullivan et al. 2009), neurodegeneration (Graham et al. 2000; Thompson et al. 2001; Ke and Qian 2003), and perivascular spaces (PVS) in the white matter (Charidimou et al. 2014).

As IDs reflect the presence of mineral deposition in the perforating vessel walls, they likely indicate some degree of microvessel dysfunction which may predispose to small vessel disease (SVD) brain damage later in life. IDs have been related to the drop in intelligence (IQ) between childhood and old age: those having more IDs when older, having the worse drop in IQ (Penke et al. 2012). Other indicators of low cognitive performance in youth (e.g. years of education) have also been associated with an increase in the risk of SVD imaging markers in later life (Backhouse et al. 2017). Visible perivascular spaces (PVS) are also a sign of microvessel dysfunction including increased blood brain barrier leakage (Brown et al. 2018). They also play an essential role in maintaining brain fluid balance and are thought to be part of the pathogenic pathway to the development of SVD and its associated brain damage. Moreover, serum markers of cerebral amyloid angiopathy in individuals with abundant lobal microbleeds have been associated with number of microbleeds and burden of PVS specifically in the centrum semiovale (CSO-PVS) (Charidimou et al. 2014; Ishikawa et al. 2018), prompting researchers to suggest that prominent CSO-PVS are a marker of amyloid deposition and hence related to microbleed formation (Ishikawa et al. 2018). The role of PVS in cognition in the healthy elderly in relation to declining cognition is, however, less clear. Some studies have suggested that PVS in the basal ganglia (Passiak et al. 2019; MacLullich et al. 2004) and hippocampus (Jimenez-Balado et al. 2018) are associated with cognitive decline while two meta-analyses (Hilal et al. 2018. Francis et al. 2019) have found inconsistent results. The role of, specifically, CSO-PVS in cognitive decline or impairment in adulthood has been studied in the context of small vessel disease (Benjamin et al. 2018; Yao et al. 2014), stroke/transient ischaemic attack (Hurford et al. 2014), hypertension (Uiterwijk et al. 2014), and epidemiology (i.e. population from specific geographic regions, e.g. Pomerania, Dijon, Hong Kong, Singapore) (Hilal et al. 2018) with conflicting results. Given the societal impact of dementia, the putative association between burden of PVS in different brain regions and dementia and/or dementia risk has been also explored (Francis et al. 2019; Debette et al. 2019) with inconclusive results. To the best of our knowledge the current literature lacks information of computational measures of CSO-PVS burden in community-dwelling older individuals in relation to specific cognitive domains. It is not known, therefore, whether CSO-PVS can (or not) be considered a determinant of non-pathological cognitive ageing.

Brain IDs and CSO-PVS have been separately associated with the burden of white matter hyperintensities (WMH), mainly thought to be of vascular origin, and whose progression has been linked to endothelial (Poggesi et al. 2015) and blood-brain barrier permeability dysfunction (Wardlaw et al. 2013b), related to the formation of both iron deposits and PVS burden (Valdes Hernandez et al. 2016). However, evidence of association between increase number of PVS and WMH is inconsistent and more data are needed (Francis et al. 2019). Moreover, hypercholesterolaemia, hypertension, diabetes, previous strokes and presence of cardiovascular disease have been related previously to the presence of CSO-PVS (Wardlaw et al. 2013a; Francis et al. 2019) and IDs (Valdes Hernandez et al. 2015).

In a large narrow-age cohort of community-dwelling septuagenarian, we investigate whether volumes of PVS and IDs are related to each other, and whether CSO-PVS volume and count are associated with poorer cognition than that expected for a given premorbid IQ. By seeking to answer these research questions, we also explore the role of WMH and vascular risk factors in CSO-PVS burden and how these in turn might affect cognition; seeking information that could explain the link between early life factors and greater SVD burden in later life. We hypothesise that CSO-PVS and brain ID are associated and that both, independently, have associations with cognitive function in generally healthy older people. From this cohort, previous research has reported independent associations between WMH, IDs and cognitive function (Valdes Hernandez et al. 2013; Penke et al. 2012; Valdés Hernández et al. 2015), and with other related factors (e.g. nutritional (Valdes-Hernandez et al. 2014), or vascular risk (Aribisala et al. 2014a; Valdés Hernández et al. 2015)); the mediating role of WMH on the associations between IDs and cognition (Valdes Hernandez et al. 2016), and the association of PVS, WMH and markers of inflammation (Aribisala et al. 2014b) (http://www.lothianbirthcohort.ed.ac.uk/).

## Materials and methods

**Fig. 1:**
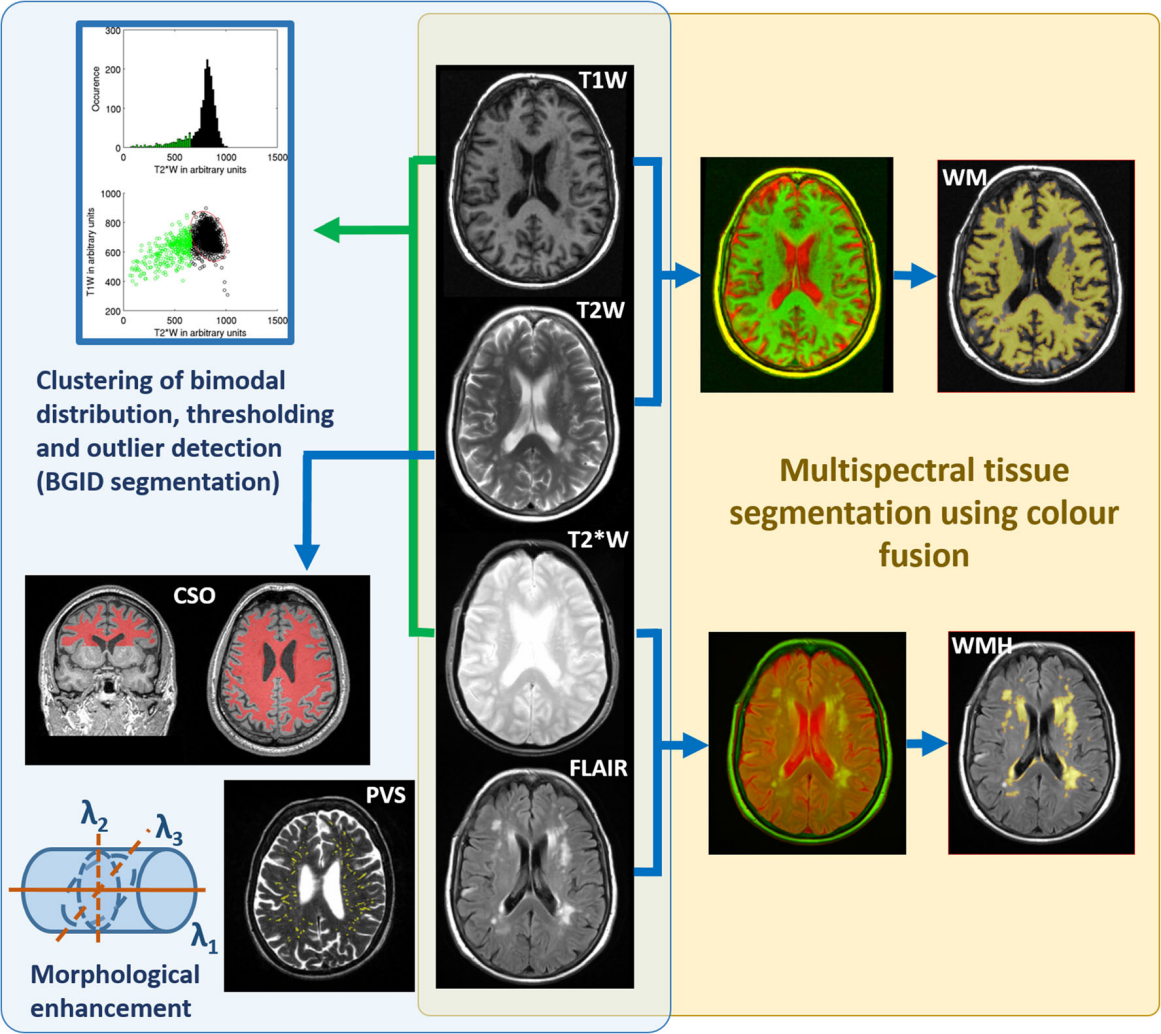
Schematic representation of the automatic (light blue) and semi-automatic (light yellow) image processing pipelines. Normal-appearing white matter (WM) masks are obtained from combining T1- and T2-weighted images (T1W and T2W respectively), whilst white matter hyperintensity (WMH) masks are obtained from the FLAIR and T2*-weighted colour combination. Brain iron deposits are obtained from thresholding the outliers of the bimodal lower cluster of the T1- and T2*-weighted images (T1W and T2*W respectively). Perivascular spaces (PVS) in the centrum semiovale (CSO) are obtained from thresholding the T2W image after enhancing the tubular-like structures in the region of interest

We analysed structural brain MRI and cognitive data from 701 community-dwelling individuals from the Lothian Birth Cohort 1936 at mean age 72.7 years (SD 0.7, range 71.1 to 74.2), from whom written informed consent was obtained under protocols approved by the Lothian (REC 07/MRE00/58) and Scottish Multicentre (MREC/01/0/56) Research Ethics Committees. From the MRI, brain IDs were assessed automatically (Glatz et al. 2015) in a pipeline that clusters the bimodal distribution of T1W and T2*W intensities in the basal ganglia and compares this distribution with that of normal appearing tissue; an adaptive threshold is then applied to obtain a robust estimate of the volume of T2*W hypointensities that might correspond to brain IDs. WMH volume was assessed semi-automatically (Hernandez et al. 2010) using a multispectral colour-fusion segmentation scheme, and CSO-PVS volume and count were obtained fully automatically (Ballerini et al. 2018) after thresholding the output from a multidimensional filter that enhances vessel-like structures (Fig. 1). All MRI data, and the automatic and semi-automatic methods used here are publicly available (see Online Methods for details). The cognitive variables analysed were general fluid intelligence (g), general information processing speed (g-speed), and general memory (g-memory), and intelligence (IQ) at age 11. They were generated using principal component analysis from batteries of well-validated cognitive tests (Penke et al. 2012). Age in days, biological sex, and self-reported vascular risk factors (VRF) (i.e. hypertension, hypercholesterolaemia, diabetes and history of cardiovascular disease and stroke) were also used in the analyses. We used MATLAB R2014a to perform linear regression and general linear models (GLMs). Variables and interaction terms for the GLMs were selected based on plausibility from published analyses. All volumetric imaging measurements used in these models were adjusted by head size. For the linear regression models, a combined VRF variable was obtained by summing all self-reported VRF by coding present = 1, not present =0. GLM and regression models used a sample size of 540, which is the number of individuals with MRI datasets that provided accurate CSO-PVS measures (i.e. volume and count). IBM SPSS Statistics (release 21.0.0.0, 2012) was used to calculate bootstrapped bivariate parametric and non-parametric cross-correlations between the imaging and cognitive variables, and generate the descriptive statistics for all variables involved in the analyses. For the correlations, cases were excluded pairwise from the 701 individuals that comprised our initial sample, and the imaging variables were also adjusted by head size. Parametric correlations between raw ID volumes and cognition, raw WMH and ID volumes, and raw WMH volumes and cognition were not calculated as they have been reported previously (Valdes Hernandez et al. 2015; Valdes Hernandez et al. 2013). All results are given with a precision of two significant decimal places. Full details of the sample, cognitive data used, MRI acquisition and processing can be found in the Online Methods.

## Results

### Sample characteristics

The descriptive statistics of the imaging, self-reported vascular risk factors and cognitive variables involved in the analyses are given in Table 1. CSO-PVS were successfully automatically assessed in 540 individuals, with numbers of PVS ranging from 41 to 847, which represent volumes from 0.24 ml to 8.28 ml (mean 3.29 ml, 0.22% of the ICV). These correspond with neuroradiological ratings in the Potter-Wardlaw scale (Potter et al. 2015) from 1 to 4 (median 2). IDs and WMH in this sample have been characterised in detail previously (Valdes Hernandez et al. 2013; Valdes Hernandez et al. 2012b). Briefly, IDs were assessed in 672/701 MRI datasets, who had the sequences required for assessing mineral deposition. From these, IDs were present in 490 participants. The median total volume of IDs in the sample (i.e. *n* = 672) was 0.04 ml (0.003% of the ICV), and the median load in those individuals who had them was 0.10 ml (0.007% of the ICV) (IQR 0.25 ml), ranging from 0.0020 ml to 3.22 ml. The WMH median volume in this sample was 7.7 ml (0.53% of the ICV) (IQR = 13.33 ml) (Table 1). Given the known association between biological sex and brain ID burden (Valdes Hernandez et al. 2015) and reports that considered sex differences, men may have greater PVS volume than women, particularly in the white matter (Ramirez et al. 2015); thus descriptive statistics are given for each biological sex group. However, only the median and distribution of PVS count significantly differed between men (median: 440, IQR: 229) and women (median: 375, IQR: 174) in this sample (*p* = 0.002 and *p* < 0.001 respectively). The number of men and women in the sample was balanced. The number of men with diabetes and history of cardiovascular disease was approximately double the number of women with these VRFs. This biological sex difference was not observed for hypertension, hypercholesterolaemia and previous strokes.

**Table 1:** Imaging volumetric measures, vascular risk factors and cognitive variables considered in the analyses. Values reported are mean (standard deviation) unless indicated otherwise

Parameter	n	Men (*n* = 373)	Women (*n* = 328)	Total (*n* = 701)
g factor	696	−0.020 (1.074)	0.054 (0.93)	0.014 (1.01)
g-speed	686	−0.047 (1.064)	0.058 (0.96)	0.0031 (1.02)
g-memory	681	−0.094 (1.073)	0.11 (0.95)	0.0025 (1.02)
Age 11 IQ	663	98.86 (16.52)	102.73 (13.73)	100.68 (15.39)
MMSE	700	28.56 (1.59)	28.98 (1.16)	28.76 (1.42)
Total ID vol (ml)**†**	672	0.046 (0.20)	0.036 (0.18)	0.040 (0.20)
CSO-PVS vol (ml)	540	3.41 (1.56)	3.14 (1.33)	3.29 (1.46)
WMH vol (ml)**†**	673	7.87 (13.22)	7.38 (13.83)	7.70 (13.33)
Hypertension (n(%))	701	184 (26.25)	157 (22.35)	341 (48.6)
Diabetes (n(%))	701	53 (7.56)	24 (3.44)	77 (11)
Hypercholesterolaemia (n(%))	701	156 (22.25)	134 (19.25)	291 (41.5)
CVD (n(%))	701	124 (17.69)	67 (9.51)	191 (27.2)
Stroke (n(%))	700	69 (9.86)	55 (7.84)	124 (17.7)

Legend: n: valid sample size, WMH: white matter hyperintensities, ID: iron deposition, CSO-PVS: perivascular spaces in the centrum semiovale and corona radiata supraventricular, CVD: history of cardiovascular disease, (**†**): median (interquartile range) values as variables were not normally distributed

### Bivariate relationships

**Fig. 2:**
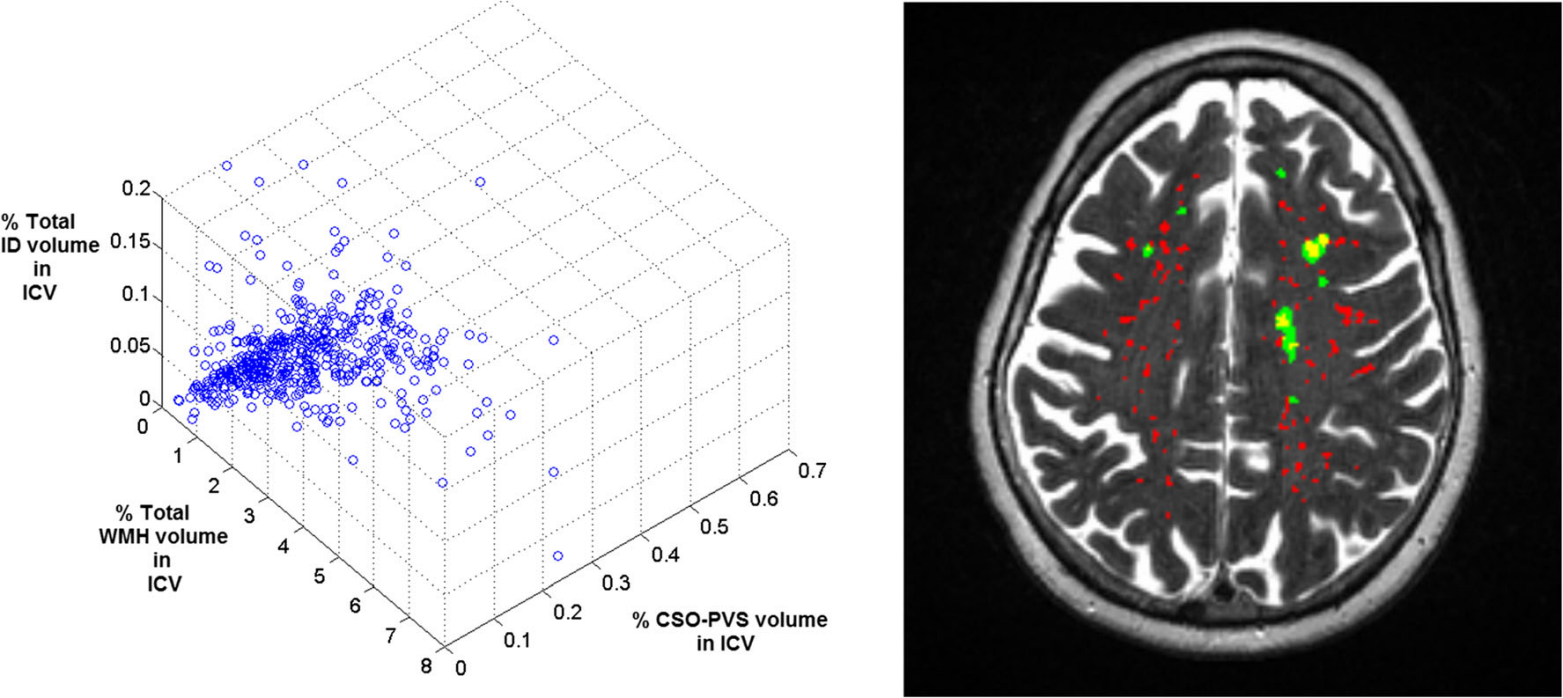
3D representation of the distribution of total iron deposition, white matter hyperintensity and centrum semiovale perivascular spaces volumes adjusted by intracranial volume (ICV) in the sample (left) and axial T2-weighted slice showing the perivascular spaces (red), white matter hyperintensities (green) and their overlap (yellow)

The cross-correlation matrix showing the bootstrapped bivariate associations among the cognitive ability measures and brain variables adjusted for head size is shown in Table 2. The percentage of total ID volume in ICV was negatively correlated with general cognition (Spearman ρ = −0.090, *p* = 0.049). CSO-PVS volume (also adjusted for head size) was negatively correlated with general cognition (Spearman ρ = −0.095, *p* = 0.036), g-memory (Spearman ρ = −0.10, *p* = 0.029) and g-speed (Spearman ρ = −0.11, *p* = 0.017). However, CSO-PVS count was not related with the cognitive variables. The percentage of CSO-PVS volume in ICV and PVS count were correlated with the other two imaging measurements adjusted by head size: ID volumes (Spearman ρ = 0.13 and 0.14 p < 0.001) and WMH volume (Spearman ρ = 0.48 and 0.19 respectively, p < 0.001) (Fig. 2, Table 2). The percentages of WMH and ID volumes in ICV were not correlated.

**Table 2:** Bivariate pairwise cross-correlations between the cognitive and imaging variables evaluated. Spearman (ρ) values are given in the triangle below the main diagonal and Pearson’s (r) values are given in the upper triangle above the main diagonal. The significance level is indicated as follows: * *p* < 0.05, ** *p* < 0.001. All results are given with a precision of two significant decimal places

	g factor	g-speed	g-memory	Age 11 IQ	%Total ID vol. in ICV (†)	%CSO-PVS volume in ICV	CSO-PVS count	%WMH vol. in ICV (†)
g factor	1	0.76**	0.70**	0.60**	−0.079	−0.11*	−0.036	−0.18**
g-speed	0.74**	1	0.50**	0.42**	−0.066	−0.12**	−0.041	−0.20**
g-memory	0.68**	0.46**	1	0.55**	−0.071	−0.11*	−0.082	−0.14**
Age 11 IQ	0.56**	0.39**	0.54**	1	−0.0060	−0.057	−0.045	−0.048
%Total ID vol. in ICV (†)	−0.090*	−0.077	−0.087	−0.016	1	0.052	0.066	0.022
%CSO-PVS volume in ICV	−0.095*	−0.11*	−0.10*	−0.067	0.13**	1	0.82**	0.43**
CSO-PVS count	−0.011	−0.033	−0.060	−0.033	0.14**	0.83**	1	0.088
%WMH volume in ICV (†)	−0.18**	−0.25**	−0.14**	−0.058	0.039	0.49**	0.19**	1

Legend: (†) Not normally distributed data

### General linear models and linear regression models

#### Association between CSO-PVS measures and total ID volume

GLM revealed that the percentage of CSO-PVS volume in ICV was not associated with the percentage of total ID volume in ICV but with the load of WMH (B = 0.051 *p* < 0.01). The same GLM, but using instead CSO-PVS count, yielded similar results with the association with WMH volume (B = 22.85 p < 0.01). However, in both models, CSO-PVS measures were associated with interaction factors between the percentage of ID volume in ICV, various VRF, and the percentage of WMH volume in ICV (Table 3). WMH were observed around CSO-PVS in many cases (Fig. 2).

**Table 3:** General linear models

Model no.	Outcome	Predictors (in addition to age and biological sex)				Interaction terms			
	name	name	B	SE	*p* value	name	B	SE	p value
1	%CSO-PVS vol. in ICV	% ID vol. in ICV	0.12	0.31	0.69	ID vol · WMH vol	−0.39	0.15	0.011
		% WMH vol. in ICV	0.051	0.0052	1.11e-20	ID vol · hypertension	1.083	0.36	0.0029
		hypertension	−0.0065	0.0092	0.48	ID vol · hypercholest.	−0.59	0.32	0.065
		diabetes	0.0061	0.013	0.64	ID vol · hist of CVD	−0.88	0.37	0.019
		Hypercholesterolaemia	−0.0037	0.0094	0.70	ID vol · prev stroke	0.77	0.38	0.042
		History of CVD	0.011	0.010	0.25				
		Previous stroke	−0.017	0.012	0.15				
2	CSO-PVS count	% ID vol. in ICV	15.69	518.43	0.97	ID vol · WMH vol	−371.46	256.13	0.15
		% WMH vol. in ICV	22.85	8.61	0.0082	ID vol · hypertension	1709.10	600.60	0.0046
		hypertension	−20.085	15.33	0.19	ID vol · hypercholest.	−1047.30	532.46	0.049
		diabetes	28.23	21.77	0.19	ID vol · hist of CVD	−1052.90	621.21	0.091
		Hypercholesterolaemia	2.51	15.67	0.87	ID vol · prev stroke	1323.70	628.74	0.036
		History of CVD	17.37	16.62	0.30				
		Previous stroke	−39.77	19.93	0.046				
3	g-factor	% CSO-PVS vol. in ICV	−83.65	45.22	0.065	CSO-PVS vol · age	1.15	0.62	0.065
		% WMH vol. in ICV	−0.15	0.052	0.0037	CSO-PVS vol · hist of CVD	−0.32	0.98	0.74
		hypertension	−0.19	0.091	0.034	CSO-PVS vol · diabetes	−1.33	1.37	0.33
		diabetes	0.14	0.35	0.68				
		Hypercholesterolaemia	−0.012	0.094	0.90				
		History of CVD	0.0043	0.24	0.98				
		Previous stroke	−0.13	0.11	0.26				
4	g-factor	CSO-PVS count	−0.044	0.029	0.13	CSO-PVS count · age	0.00061	0.00041	0.13
		% WMH vol. in ICV	−0.16	0.048	0.00086	CSO-PVS count · hist of CVD	−0.00029	0.00065	0.66
		hypertension	−0.19	0.091	0.033	CSO-PVS count · diabetes	−0.0012	0.00087	0.15
		diabetes	0.39	0.41	0.35				
		Hypercholesterolaemia	−0.020	0.094	0.83				
		History of CVD	0.063	0.30	0.83				
		Previous stroke	−0.12	0.11	0.30				
5	g-speed	% CSO-PVS vol. in ICV	−56.35	45.80	0.22	CSO-PVS vol · age	0.77	0.63	0.22
		% WMH vol. in ICV	−0.18	0.053	0.00087	CSO-PVS vol · hist of CVD	−0.14	0.99	0.89
		hypertension	−0.13	0.092	0.14	CSO-PVS vol · diabetes	−1.75	1.38	0.21
		diabetes	0.15	0.35	0.66				
		Hypercholesterolaemia	−0.11	0.095	0.25				
		History of CVD	−0.019	0.25	0.94				
		Previous stroke	−0.081	0.11	0.48				
6	g-speed	CSO-PVS count	−0.037	0.030	0.22	CSO-PVS count · age	0.00051	0.00041	0.22
		% WMH vol. in ICV	−0.19	0.048	7.27e-05	CSO-PVS count · hist of CVD	0.00061	0.00066	0.36
		hypertension	−0.13	0.092	0.15	CSO-PVS count · diabetes	−0.0014	0.00088	0.12
		diabetes	0.34	0.42	0.42				
		Hypercholesterolaemia	−0.10	0.095	0.29				
		History of CVD	−0.31	0.30	0.31				
		Previous stroke	−0.063	0.11	0.58				
7	g-memory	% CSO-PVS vol. in ICV	−114.46	48.35	0.018	CSO-PVS vol · age	1.57	0.67	0.019
		% WMH vol. in ICV	−0.13	0.056	0.020	CSO-PVS vol · hist of CVD	0.15	1.045	0.88
		hypertension	0.00035	0.097	0.99	CSO-PVS vol · diabetes	−1.96	1.46	0.18
		diabetes	0.16	0.37	0.67				
		Hypercholesterolaemia	−0.064	0.10	0.52				
		History of CVD	−0.0095	0.26	0.97				
		Previous stroke	−0.16	0.12	0.20				
8	g-memory	CSO-PVS count	−0.030	0.031	0.34	CSO-PVS count · age	0.00040	0.00043	0.35
		% WMH vol. in ICV	−0.14	0.051	0.0048	CSO-PVS count · hist of CVD	0.00053	0.00070	0.45
		hypertension	0.0022	0.097	0.98	CSO-PVS count · diabetes	−0.0019	0.00093	0.041
		diabetes	0.54	0.44	0.22				
		Hypercholesterolaemia	−0.073	0.10	0.47				
		History of CVD	−0.18	0.32	0.56				
		Previous stroke	−0.13	0.12	0.29				

#### Association between CSO-PVS measures and cognitive indicators

As per GLMs, the percentage of CSO-PVS volume in ICV and its interaction with age were associated with g-memory. The interaction of CSO-PVS count with diabetes was also associated with g-memory (Table 3). For each cognitive measure as an outcome variable, two linear regression models that used the CSO-PVS volume adjusted by ICV as the dependent variable, together with age, biological sex, percentage of WMH volume in ICV and the combined VRF variable as covariates, were used to re-evaluate the putative associations between CSO-PVS volumes and cognitive indicators. One of these models included age 11 IQ as covariate and the other did not. Linear regression models could not confirm that the CSO-PVS volume as percentage in ICV was associated with any of the three cognitive measures analysed. From these models, the percentage of WMH volume in ICV was the imaging parameter negatively associated with all cognitive variables, with strength and significance levels similar to those reported previously using structural equation models (Valdes Hernandez et al. 2013). The same re-evaluation was done using, instead, as independent variable, the number of CSO-PVS counted yielding similar results.

## Discussion

CSO-PVS volume, despite being correlated with the three cognitive domains, was only weakly associated with g-memory in this sample after accounting for age, biological sex, white matter hyperintensity burden and VRFs in a model that also considered the interactions of CSO-PVS burden with age, cardiovascular risk factors and diabetes. From these models, the interaction of the number of CSO-PVS with diabetes and the volume of CSO-PVS with age were also associated with g-memory. However, these associations could not be reproduced on linear regression models, which did not account for the interactions previously mentioned but evaluated the possible influence of childhood intelligence. Thus, we can conclude that in our cohort 1) the burden of CSO-PVS has no direct association with general cognition at older age, and 2) the possible effect that the CSO-PVS burden could have in general memory at older age could be moderated by the presence (or not) of diabetes and/or by age.

The first conclusion is in-line with those reported by other studies that have explored the possibility of an association of PVS burden with cognition in older people. A previous study in a healthy ageing sample (100 men) reported a correlation of PVS visual ratings in the basal ganglia and centrum semiovale with specific cognitive tests: non-verbal reasoning and general visuospatial ability (MacLullich et al. 2004). A population-based study on 1778 non-demented participants from 65 to 80 years of age with a 4 year follow-up, concluded that baseline PVS assessments were not associated with baseline cognitive performance (Zhu et al. 2010). Although the same study found that higher rating scores of PVS in the basal ganglia were associated with cognitive decline after 4 years, this was not the case for PVS scores in the white matter (i.e. CSO). Studies have found associations between higher PVS scores in the basal ganglia and poorer general cognition (Huijts et al. 2014; Hurford et al. 2014), but not between the latter and either total or CSO PVS (i.e. visual scores, volumes or count) (Benjamin et al. 2018; Molad et al. 2017). A meta-analysis of the literature (Francis et al. 2019) only found one study (Uiterwijk et al. 2014) that reported an association between CSO-PVS and cognition, but with borderline significance. Systematic literature reviews have not found enough evidence to conclude on the possible association between CSO-PVS and dementia or dementia risk (Francis et al. 2019; Debette et al. 2019). Our results add to the hypotheses that basal ganglia and CSO might have different underlying small vessel arteriopathies to which PVS are functionally linked (Hurford et al. 2014). However, the variability in the associations reported between PVS and cognition could be partly due to studies having used PVS visual rating scores, and the relatively lower sensitivity or perhaps higher heterogeneity of the visual rating scales applied. Robust computational methods to quantitatively assess PVS are emerging (Feldman et al. 2018; Dubost et al. 2019) in addition to the method applied in this work (Ballerini et al. 2018). Cross-validation of their results in different cohorts through a coordinated international effort is now needed.

The second conclusion adds to the current knowledge on the possible effects that the presence of CSO-PVS could have on general memory in older age and opens an avenue of research on mechanisms underlying the progression of CSO-PVS. A large study of 1818 stroke- and dementia-free participants, which assessed PVS specifically in the hippocampus, a brain structure known to be associated with memory, failed to find any association between the burden of PVS in this structure and baseline cognitive performance or incident dementia in the 8-year follow-up (Yao et al. 2014). However, this study did not assess PVS in the centrum semiovale and neither the possible moderator role of diabetes, present only in 8% of the sample. Moreover, it did not evaluate a general memory score, but only visual memory. Risk factors for the presence of PVS differ in various brain regions (Zhang et al. 2014). The burden of WMH, known to have a strong negative association with cognition, has been cited as the main risk factor for CSO-PVS (Zhang et al. 2014). In our sample, the total volume of WMH was the only covariate consistently associated with both CSO-PVS and the three cognitive measures, an association that was explored in detail previously (Valdes Hernandez et al. 2013), and which has also been reported in patients with cerebral small vessel disease (SVD) (Benjamin et al. 2018; Molad et al. 2017). As well as increasing age, Type 2 diabetes is known to be associated with higher WMH burden (Tamura and Araki 2015). The inter-relations between WMH burden, CSO-PVS volume and count, age, impaired glucose and insulin transfer mechanisms and memory, although have been independently explored, warrant further research.

Our hypothesis that CSO-PVS would be associated with ID was partly prompted by studies that found associations between serum markers of cerebral amyloid angiopathy, number of microbleeds and CSO-PVS burden (Charidimou et al. 2014; Ishikawa et al. 2018). A study on equal-sized samples of Alzheimer’s disease (AD) patients and patients diagnosed with vascular cognitive impairment reported CSO-PVS being associated with AD and not with amyloid burden - a pathological finding characteristic of AD - (Banerjee et al. 2017). We obtained statistically significant non-parametric correlations between CSO-PVS and total ID loads, but subsequent evaluations showed no direct association between these imaging markers. Interestingly, the number of CSO-PVS was associated with the interaction between ID volume adjusted by head size and hypercholesterolaemia. In our cohort, IDs in the corpus striatum, not brain microbleeds, determine the brain ID burden (Valdes Hernandez et al. 2015), and they have been found associated with hypercholesterolaemia (Valdes Hernandez et al. 2015).

As the actual volume of iron accumulation in tissues cannot be accurately determined using structural MRI techniques (Valdés Hernández et al. 2015), our analyses are based on volumetric measurements that, although accurate, reflect the effect that iron particles in brain tissue have on the MR signal. This is partly affected by the susceptibility of the metal/metalloid particles influenced by their aggregation, proportion and interaction with the underlying tissue among other factors (Glatz et al. 2013) and merits more research. Due to the low incidence of microbleeds in this cohort we did not analyse them separately. Replication of our analyses on cohorts with higher prevalence of this type of iron deposition is, therefore, necessary. Another limitation is that it was possible to obtain valid quantitative measures of CSO-PVS burden in only 77% of the available datasets, mainly due to motion artefacts. Although the fully automatic nature of these PVS measures makes them robust against inter−/intra-observer variations, the analyses were based on data from around 30% fewer individuals than the recruited sample. Also, given that we analysed a year-of-birth cohort, we could not fully explore the influence of age. However, the association between increasing age and higher PVS burden in both: basal ganglia and CSO, is well reported in the present literature (Francis et al. 2019; Hilal et al. 2018; Debette et al. 2019).

However, despite these limitations, this paper adds to the literature investigating the role that CSO-PVS play on cognition and their association with brain ID in a large cohort representative of “healthy ageing”, the latter being explored here for the first time. Amongst its strengths are the use of state-of-the-art quantitative methods to assess CSO-PVS, IDs and WMH volumes. Our study has both scientific and practical values, adding information related to the putative mechanisms underlying cognition, which could, in turn, help designing interventions aimed at successful cognitive ageing. Our sample is representative of the ID distribution, CSO-PVS and WMH load of community-dwelling septuagenarian Caucasian individuals, being our study relevant for epidemiological and ageing studies.

## Electronic supplementary material

ESM 1 (DOCX 28 kb)
