# Supplementary material for: Perivascular spaces in the centrum semiovale at the beginning of the 8th decade of life: effect on cognition and associations with mineral deposition
Source: Brain Imaging Behav. 2019 Jun 27;14(5):1865–75. doi: 10.1007/s11682-019-00128-1 (PMC7572330; doi:10.1007/s11682-019-00128-1)
Supplement: Supplementary file 1 — (DOCX 28 kb) [file 11682_2019_128_MOESM1_ESM.docx]

**Perivascular spaces in the centrum semiovale at the beginning of the 8^th^ decade of life: effect on cognition and associations with mineral deposition**

**Online Methods**

**Subjects**

The Lothian Birth Cohort 1936 (LBC1936) comprises mostly healthy community-dwelling older people mostly living in the Edinburgh area of Scotland. All were born in 1936 and most took an intelligence test at age 11 years in the Scottish Mental Survey of 1947[1]. At mean age 70 1091 individuals were recruited to a study of cognition in normal ageing. Of these, 866 repeated the same cognitive tests three years later and 700 (328 females and 372 males) had a brain MRI scan three years later mean age 72.7 years (SD 0.7, range 71.1 to 74.3). From the 700 brain image sets, 486 had the T2-weighted (T2W) MRI sequence with the quality required to computationally and fully automatically assess perivascular spaces. Written informed consent was obtained from all participants under protocols approved by the Lothian (REC 07/MRE00/58) and Scottish Multicentre (MREC/01/0/56) Research Ethics Committees.

**MRI scans**

MRI scans were acquired using a 1.5T GE Signa Horizon HDxt clinical scanner (General Electric, Milwaukee, WI, USA) operating in research mode using a self-shielding gradient set with maximum gradient of 33 mT/m and an 8-channel phased-array head coil. The imaging protocol is fully described elsewhere[2]. For this particular study, we used data obtained from processing coronal T1-weighted (T1W) volumes acquired with a 3D inversion recovery prepared fast gradient echo sequence (TR/TE/TI = 9.7/3.984/500 ms, flip angle α = 8°, bandwidth 15.63 kHz, voxel size 1x1x1.3 mm^3^), axial T2W volumes acquired with a fast spin-echo sequence (TR/TE = 11320/102 ms, bandwidth 20.83 kHz, voxel size 1x1x2 mm^3^), axial FLAIR volumes (TR/TE/TI = 9000/140/2200 ms, bandwith15.63 kHz, voxel size 1x1x4 mm^3^), and axial T2*W volumes acquired with a 2D gradient-echo sequence (GRASS, TE/TR = 15/940 ms, flip angle α = 20°, bandwidth 12.5kHz, voxel size 1x1x2 mm^3^). None of these sequences had interslice gap and, for all, the field-of-view (FOV) in the acquisition plane was 256x256 mm^2^. All image data is available through the BRAINS image database (<https://www.brainsimagebank.ac.uk/>).

**Image processing**

Multifocal T2*W hypointensities in the corpus striatum were assessed fully automatically using the method described in Glatz et al. (2014)[3], and freely available at <https://github.com/aglatz/mineral-deposit-segmentation-pipeline/tree/master/libBRIC/mineral-deposit-segmentation>. T2*W hypointensities were separately identified, extracted and quantified in the pre-processed T2*W images semi-automatically using the ‘Object Counter’ module in Analyze^TM^ 10.0 following a validated procedure[4,5]. Intracranial volume (ICV) and WMH volume were segmented as described in the study protocol[2] using MCMxxxVI_ALE, a software tool freely available from ([www.sourceforge.net/projects/bric1936](http://www.sourceforge.net/projects/bric1936)). All these measurements were visually checked and manually rectified if needed.

Perivascular spaces in the centrum semiovale and deep corona radiata supraventricular were segmented on the T2W images by a fully automatic pipeline[6] that uses the visual neuroradiological ratings as input to an ordered logit model to optimise the parameters of a directional filter[7] that enhances the PVS (or like) bright and elongated structures in the region of interest on T2W. The region of interest was segmented by warping the relevant atlas (<http://datashare.is.ed.ac.uk/handle/10283/2216>) to the native T2W space, for which NiftyReg from <http://sourceforge.net/projects/niftyreg/> was applied using TractoR (<http://www.tractor-mri.org.uk/diffusion-processing>).

**Cognitive testing and cognitive variables**

For this work, we used cognitive measures obtained at the time of MRI scanning (mean age 72.7, SD 0.7 years). These cognitive variables[1] were: the general cognitive components of fluid intelligence (g), general processing speed (g-speed) and general memory (g-memory). These general cognitive ability measures were generated using principal component analysis from batteries of well-validated cognitive tests as described in [8]. To derive g, six subtests of the WAIS-III^UK[9]^ were used: Digit Symbol, Digit Span Backward, Symbol Search, Letter-Number Sequencing, Block Design and Matrix Reasoning. g-memory was derived from five subtests from the WMS-III^UK[10]^: Logical Memory Total Immediate and Delayed Recall, Verbal Paired Associates Immediate and Delayed Recall, and Spatial Span Total Score; and two subtests from the WAIS-III^UK^: Letter-Number Sequencing and Digit Span Backward. g-speed was obtained from two reaction time tests (Simple Reaction Time and Choice Reaction Time), an Inspection Time test, and two WAIS-III^UK^ subtests: Digit Symbol and Symbol Search. We also used the cognitive ability at age 11, assessed using the Moray House Test IQ score from the Scottish Mental Survey of 1947, which is considered a good measure of general intelligence[11].

Vascular risk factors

Vascular and health factors were collected during a medical interview also at the time of the MRI scanning. Study participants disclosed whether they had received a diagnosis of hypertension, high cholesterol, or diabetes, their history of cardiovascular disease and previous strokes. Presence of each self-reported factor was coded as 1 (0 denoted absent). An aggregate score of contemporaneous vascular risk was derived from these factors and the presence (1)/absence (0) of old infarcts identified on the MRI scan[12].

**Statistical analyses**

The total iron, CSO-PVS and WMH volumes were standardised by ICV to account for inter-individual differences in head size without the confounding effect of global brain atrophy. Age in days at the time of MRI scanning and/or cognitive testing was used as a covariate in all models. We used MATLAB R2014a to construct backwards step-wise general linear models to evaluate: 1) Influence of CSO-PVS volume and count in cognition accounting for age, gender, WMH volume, childhood intelligence and vascular risk factors, and 2) Association between CSO-PVS volume and count and total ID volume accounting for age, gender, vascular risk factors and WMH volume. We appraised the model fitness using the *p*-value for the chi-squared test of the change in the deviance whilst adding or removing terms. The model with all possible interactions was the largest model to consider in the evaluation. Multivariate linear models were used to validate/explore the generalisation of the results against possible over-fitness in the step-wise general linear models.

Reference List

[1] Deary,IJ, Gow,AJ, Taylor,MD, Corley,J, Brett,C, Wilson,V et al. The Lothian Birth Cohort 1936: a study to examine influences on cognitive ageing from age 11 to age 70 and beyond. *BMC Geriatr* 2007; **7**: 28.

[2] Wardlaw,JM, Bastin,ME, Valdes Hernandez,MC, Munoz Maniega,S, Royle,NA, Morris,Z et al. Brain aging, cognition in youth and old age and vascular disease in the Lothian Birth Cohort 1936: rationale, design and methodology of the imaging protocol. *Int J Stroke* 2011; **6**: 547-559.

[3] Glatz,A, Bastin,ME, Kiker,AJ, Deary,IJ, Wardlaw,JM, and Valdes Hernandez,MC. Automated segmentation of multifocal basal ganglia T2*-weighted MRI hypointensities. *Neuroimage* 2015; **105**: 332-346.

[4] Valdes Hernandez,MC, Jeong,TH, Murray,C, Bastin,ME, Chappell,FM, Deary,IJ et al. Reliability of two techniques for assessing cerebral iron deposits from structural MRI. *J Magn Reson Imaging* 2011; **33**: 54-61.

[5] Valdes Hernandez,MC, Glatz,A, Kiker,AJ, Dickie,DA, Aribisala,BS, Royle,NA et al. Differentiation of calcified regions and iron deposits in the ageing brain on conventional structural MR images. *J Magn Reson Imaging* 2014; **40**: 324-333.

[6] Ballerini,L, Lovreglio,R, Valdés Hernández,MC, Ramirez,J, MacIntosh,B, Black,S et al. Perivascular Spaces Segmentation in Brain MRI Using Optimal 3D Filtering. *Sci Rep* 2018; **8**: 2132.

[7] Frangi,AF, Niessen,WJ, Vincken,KL, and Viergever,MA. Multiscale vessel enhancement filtering. 1998; **1**: 130-137.

[8] Penke,L, Valdes Hernandez,MC, Muñoz Maniega,S, Gow,AJ, Murray,C, Starr,JM et al. Brain iron deposits are associated with general cognitive ability and cognitive aging. *Neurobiol Aging* 2012; **33**: 510-551.

[9] Wechsler,D. WAIS-III^UK^ administration and scoring manual. 1998;

[10] Wechsler,D. WMS-III^UK^ administration and scoring manual. 1998;

[11] Deary,IJ, Whiteman,MC, Starr,JM, Whalley,LJ, and Fox,HC. The impact of childhood intelligence on later life: following up the Scottish mental surveys of 1932 and 1947. *J Pers Soc Psychol* 2004; **86**: 130-147.

[12] Valdes Hernandez,M, Booth,T, Murray,C, Gow,A, Penke,L, Morris,Z et al. Brain white matter damage in aging and cognitive ability in youth and older age. *Neurobiol Aging* 2013; **34**: 2740-2747.
